# Prevalence of diabetic retinopathy and self-reported barriers to eye care among patients with diabetes in the emergency department: the diabetic retinopathy screening in the emergency department (DRS-ED) study

**DOI:** 10.1186/s12886-022-02459-y

**Published:** 2022-05-27

**Authors:** Andrew M. Williams, Jared M. Weed, Patrick W. Commiskey, Gagan Kalra, Evan L. Waxman

**Affiliations:** 1 Department of Ophthalmology, University of Pittsburgh Medical Center, 203 Lothrop Street, 8th floor, Pittsburgh, PA 15213 USA; 2 Government Medical College & Hospital, Chandigarh, India

**Keywords:** Diabetic retinopathy, Teleophthalmology, Telemedicine, Digital fundus photography, Screening, Barriers to eye care, Emergency department

## Abstract

**Background:**

Screening for diabetic retinopathy (DR) is suboptimal, and patients with diabetes who present to the emergency department (ED) may be at particularly high risk of undiagnosed DR. The purpose of this study is to determine the prevalence of DR among diabetic patients who present to the ED of our tertiary medical center using teleophthalmology and to assess self-reported barriers to eye care.

**Methods:**

This cross-sectional, single-institution study recruited clinically stable diabetic patients who presented to the ED during daytime hours over 29 total weekdays across 2 months in 2018 and 2019. Participants had nonmydriatic, 45-degree, single-field digital retinal photographs taken on site (Digital Retinal System, Centervue). Following retinal imaging, participants then completed a survey about barriers to regular eye care and their acceptance of potential interventions to promote screening. Digital retinal photographs were interpreted remotely by a board-certified ophthalmologist and communicated to participants’ primary care physician and/or endocrinologist.

**Results:**

Over the study period, 275 ED patients had a documented diagnosis of diabetes, of whom 167 were deemed clinically stable for the study and 141 were invited to participate. Sixty-four were enrolled, of whom 50 had gradable-quality fundus images (78%). Of these 50 patients, almost all had type 2 diabetes (47, 94%), with an average disease duration of 12 ± 9 years and mean hemoglobin A1c of 8.1 ± 2.0% (mmol/mol). Based on fundus photography, 14 patients (28%) were diagnosed with DR, which was newly diagnosed for 10 (20% of the total study population). Severity was most commonly mild or moderate (12/14, 86%), with 1 case of severe nonproliferative DR and 1 proliferative DR. The majority (26, 52%) reported at least one barrier to routine eye care in our self-administered survey, of which having too many appointments (6, 12%) and cost (5, 10%) were frequently cited as most important. The majority were receptive to interventions to promote DR screening, including reminder phone calls (29, 58%) and text messages (28, 56%).

**Conclusions:**

Digital fundus photography in the ED detected a high rate of undiagnosed DR. Half of participants reported barriers to routine care, and most were receptive to messaging interventions to schedule an eye exam. Future studies are warranted to assess scalability of ED-based screening programs and their follow-through rates.

## Background

Diabetic retinopathy (DR) is a potentially blinding complication of diabetes mellitus that affects about 28% of diabetics in the United States [1]. DR can typically be detected with annual screening, but many Americans with diabetes mellitus do not receive routine surveillance to prevent visual impairment or blindness [2, 3]. In fact, only about 60% of patients with diabetes report having had a dilated eye exam in the last year [4]. Undetected DR may be particularly prevalent in hospital settings, where we have previously found a DR prevalence of 44% among diabetic inpatients, over half of whom were previously undiagnosed [5].

Low uptake of diabetic screening exams may be mitigated by teleophthalmology, by which retinal images can be acquired outside of eye clinics and interpreted by ophthalmologists remotely. Offering retinal imaging outside of eye clinic settings could expand access to DR screening to patients who may otherwise go unexamined. For instance, teleophthalmology for DR screening in primary care offices has improved adherence to screening guidelines [6–8]. However, little work has been done to assess the feasibility of teleophthalmology for DR screening in hospital settings.

Emergency departments (EDs) could be a valuable setting to screen for DR. First, ED visits have been identified as a “red flag” for poor diabetes care. Specifically, diabetic patients who present to the ED are half as likely to have annual dilated fundus exams than those who do not [9]. Second, digital fundus photography in the ED is at least as effective as direct ophthalmoscopy and is feasible to conduct in the ED setting [10, 11]. Third, diabetic patients in the ED may benefit from behavioral interventions, such as reminder messages, to promote DR screening rates [12]. However, the acceptance of these interventions in this population is largely unknown. The ED provides a high-risk diabetic patient population for screening and a setting with previously validated methods of digital fundus photography [10, 11]. The purpose of this study is to determine the prevalence of DR in the ED setting, to survey barriers to regular ophthalmic care, and to assess patient willingness to engage in behavioral interventions that promote outpatient follow up.

## Methods

This prospective, cross-sectional study received approval from the University of Pittsburgh Medical Center Institutional Review Board, adhered to the tenets of the Declaration of Helsinki, and maintained compliance with the Health Insurance Portability and Accountability Act of 1996.

Patients were recruited from the ED of our tertiary academic medical center during business hours over the course of 29 days in 2018 and 2019. Study investigators reviewed the electronic record of patients with active encounters in the ED to assess for eligibility. All patients with a documented diagnosis of type 1 or 2 diabetes mellitus who presented in stable condition were included. For patients who met inclusion criteria, study investigators contacted the emergency care providers in the ED to assess patient willingness to participate in the study.

For patients interested in participating, investigators obtained informed consent, administered a study questionnaire, and obtained fundus photographs. The questionnaire, supplemented by review of the medical record, was used to document demographic information, past medical history, duration of diabetes, and hemoglobin A1c (HbA1c) level. We also inquired about past eye exams, barriers to care, and willingness to receive interventions to improve follow up.

Following administration of the study questionnaire, fundus photography was obtained in the ED by a resident physician (JMW or PWC) using a portable digital fundus camera (Digital Retinal System, Centervue). Images included a nonmydriatic, nonstereoscoptic, 45-degree, single-field image of the posterior pole of each eye, which included the macula, major vascular arcades, and the optic nerve. All patients received education about DR, the potential effects of diabetes on vision, and the importance of having regular eye examinations.

Retinal images were uploaded into the electronic health record and forwarded to a board-certified ophthalmologist (ELW) for interpretation. Patients found to have any level of DR by Early Treatment of Diabetic Retinopathy Study (ETDRS) criteria were advised to have a formal eye exam within about 2 weeks after review of the fundus photograph. Letters with the results of the screen were sent to the patient’s primary care physician and/or endocrinologist as listed in the medical record or provided by the patient.

## Results

Electronic health records of 1,404 ED encounters were reviewed, of which 275 (20%) met inclusion criteria with a documented diagnosis of diabetes. Of these 275 patients, 108 (39%) were excluded because they were determined to be clinically unstable. An additional 26 patients were unavailable for the study due to active clinical care by emergency providers or presence at an imaging study.

Of the 141 diabetic patients approached, 34 (23%) declined because they already had an eye care provider either treating DR (15/34) or screening for it (19/34), and 7 (5%) declined to participate due to denial of diabetes diagnosis. An additional 39 (28%) wished to participate but were unable to sit at the digital fundus camera due to body habitus, medical condition, or feeling unwell, and 10 (7%) were unable to complete the study due to digital fundus camera malfunction. Of the 64 patients enrolled, 14 had fundus photos of a quality too poor to analyze. In total, 50 patients were enrolled into the study with digital fundus photos sufficient for analysis.

### Patient characteristics

The 50 patients in this study included 26 women (52%), had an average age of 56 (34 to 81), and predominantly self-identified as non-Hispanic white (32, 64%) (Table 1). Almost all had type 2 diabetes (47, 94%), and the average duration of diabetes was 12 (2 to 47) years. The mean HbA1c was 8.1 ± 2.0%, and half reported being insulin dependent. Patients had various comorbidities, most commonly hypertension (41, 82%) and hyperlipidemia (29, 58%). Sixteen (32%) reported having an endocrinologist (Table 2).

**Table 1:** Demographic characteristics of 50 patients who completed DR screening in the ED

Characteristic	*n* (%)
Age (years; mean ± SD)	
56 ± 12 (range: 34–81)	
Sex	
Female	26 (52)
Male	24 (48)
Race	
African American	18 (36)
Non-Hispanic white	32 (64)
Highest level of education	
Some high school	1 (2)
High school degree	13 (26)
GED	5 (10)
Some college	8 (16)
Trade school	6 (12)
Associate’s degree	4 (8)
Bachelor’s degree	6 (12)
Master’s degree	4 (8)
Doctorate degree	3 (6)
Currently Employed	19 (38)
Annual income (dollars; mean ± SD)	
52,000 ± 60,000 (range: 8,000–300,000)	
Health insurance	50 (100)
Marital status	
Single	19 (38)
Married	19 (38)
Divorced	9 (18)
Widowed	3 (6)

*DR* Diabetic retinopathy, *ED* Emergency department, *GED* General equivalency diploma, *SD* Standard deviation

**Table 2:** Clinical characteristics of 50 patients who completed DR screening in the ED

Characteristic	*n* (%)
Diabetes type	
Type 1	2 (4)
Type 2	47 (94)
Other	1 (2)
Diabetes duration (years; mean ± SD)	
12 ± 9 (range: 2–47)	
Hemoglobin A1c (%; mean ± SD)	
8.1 ± 2.0 (range: 5.4–14.5)	
Mean arterial pressure recorded in ED (mmHg, mean ± SD)	
102 ± 4 (range: 71–126)	
Insulin dependence	25 (50)
Smoking history	
Current	13 (26)
Former	18 (36)
Never	19 (38)
Comorbidities	
Hypertension	41 (82)
Hyperlipidemia	29 (58)
Neuropathy	23 (46)
Renal disease	21 (42)
Coronary artery disease	13 (26)
Medical providers	
Primary care physician	49 (98)
Endocrinologist	16 (32)
Nephrologist	9 (18)
Pulmonologist	6 (12)
Cardiologist	8 (16)
Gastroenterologist	4 (8)
Neurologist	3 (6)
Rheumatologist	3 (6)
Vascular surgeon	2 (4)
Psychiatrist	2 (4)
Cardiothoracic surgeon	1 (2)
General surgeon	1 (2)
Infectious disease specialist	1 (2)
Report having an ophthalmologist or optometrist	39 (78)
Last self-reported dilated fundus exam	
Within 1 year	34 (68)
Over a year ago	9 (18)
Never	5 (10)
Known history of diabetic retinopathy	7 (14)

*mmHg* Millimeters of mercury, *SD* Standard deviation

Regarding eye care, 39 (78%) indicated that they regularly see an ophthalmologist or optometrist, and most (34, 68%) reported having had a dilated fundus exam in the last year. Seven (14%) reported a known history of DR (Table 2).

Patients presented to the ED for a wide range of chief complaints, most commonly chest pain (5, 17%) or dyspnea (3, 10%) (Table 3). Sixteen (32%) were admitted to the inpatient floor from the ED.

**Table 3:** Presenting chief complaint to the emergency department for 50 patients who completed DR screening in the ED

Complaint	*n* (%)
Chest pain	5 (17)
Dyspnea	3 (10)
Eye pain	2 (7)
Foot pain and/or swelling	2 (7)
Abdominal pain	1 (3)
Abnormal lab results	1 (3)
Anemia, gastrointestinal bleed	1 (3)
Cat bite	1 (3)
Congestive heart failure	1 (3)
COPD exacerbation	1 (3)
Fever, rash	1 (3)
Hyperglycemia	1 (3)
Hypoglycemia	1 (3)
Hypotension	1 (3)
Infected fistula graft site	1 (3)
Injured foot	1 (3)
Leg swelling	1 (3)
Nausea and vomiting	1 (3)
Nausea, vomiting, and abdominal pain	1 (3)
Shoulder pain	1 (3)
Sore throat	1 (3)
Supraventricular tachycardia	1 (3)

*COPD* Chronic obstructive pulmonary disease

### Screening results

Of the 50 patients who completed the screening, 14 (28%) were determined to have DR, which was a new diagnosis for 10 (20% of the total). As described in Table 4, most DR was mild (5, 10%) or moderate (7, 14%) in severity. Sixteen of the patients enrolled were subsequently admitted to the hospital, 8 (50%) of whom had diabetic retinopathy.

**Table 4:** Diabetic retinopathy screening results of 50 patients who completed DR screening in the ED

	***n* (%)**
Diabetic retinopathy, any stage	14 (28)
Mild NPDR	5 (10)
Moderate NPDR	7 (14)
With CSME	1 (2)
Severe NPDR	1 (2)
PDR	1 (2)
New diagnosis of diabetic retinopathy	10 (20)

*CSME* Clinically significant macular edema, *NPDR* Non-proliferative diabetic retinopathy, *PDR* Proliferative diabetic retinopathy

### Survey results

Almost all patients (45, 90%) reported knowing that diabetes can affect their vision. Twelve (24%) reported that their vision interferes with their daily activities, and the mean self-reported vision quality score was 8 out of 10. The majority of participants reported at least one barrier to regular eye examinations (26, 52%; Table 5). The barriers to care cited as most important included having too many other medical appointments (6, 12% of the total), inability to afford the exam (5, 10%), and having no perceived vision problems (4, 8%). Only three participants (6%) stated that transportation was the primary barrier to eye care. The majority of patients were receptive to receiving phone calls (29, 58%) or text message reminders (28, 56%) to schedule a routine eye appointment.

**Table 5:** Self-reported barriers to regular eye examinations

Barriers	*n* (%) cited as most important	*n* (%) cited^a^
Too many other medical appointments	6 (12)	7 (14)
Cannot afford the exam or co-payment	5 (10)	6 (12)
My vision is fine	4 (8)	6 (12)
Transportation issues	3 (6)	4 (8)
I am too busy	2 (4)	7 (14)
Too difficult to get an appointment	2 (4)	3 (6)
I did not know it was important	2 (4)	4 (8)
Too sick to sit for several hours in the eye clinic	0 (0)	3 (6)
Mobility issues (e.g. wheelchair-bound)	0 (0)	2 (4)
My eyes were examined by my PCP	0 (0)	2 (4)
I do not trust doctors or the medical system	0 (0)	0 (0)
Other (not specified)	1 (2)	3 (6)
None	24 (48)	24 (48)

*PCP* Primary care physician

^a^ Patients could report more than one

## Discussion

Using teleophthalmology, we identified a 28% prevalence of DR among diabetic patients presenting to our ED for care, most of whom had not been previously diagnosed. Patients in the emergency department are more likely to have poor glycemic control and miss screening exams for DR [13, 14]. The higher likelihood of poorly controlled diabetes in this setting could make the ED a high-yield setting to detect DR and to address barriers to regular care [9].

Interestingly, our 28% prevalence of DR is more similar to the prevalence found in outpatient clinics than inpatient settings. Zhang et al. detected a 29% prevalence of DR among 6797 patients in outpatient offices, with a similar proportion of vision-threatening diabetic retinopathy. [1] We had previously found a higher prevalence of DR among 113 inpatients at our hospital, of whom 44% exhibited DR [5]. Hospitalization may indicate poorer disease control and, therefore, higher risk of DR. In fact, of the 16 patients in our study who were admitted from the ED, 8 (50%) had DR. Nonetheless, our ED screening study found that most cases of DR were previously undiagnosed and that many emergency patients do not have regular eye care. The ED may serve as a valuable setting to identify barriers to outpatient care and to provide resources to address these barriers [15].

About half of participants in our study reported at least one barrier to regular eye care. The most common barriers were having too many other medical appointments and difficulty affording the costs of vision care, which were also commonly cited among diabetic inpatients at our institution [5]. Similarly, in a separate ED-based survey on barriers to care, Tian et al. found that cost and inadequate insurance predominated as reasons diabetic patients reported for not having an annual eye exam. [14] While less commonly cited in our population, transportation issues and not knowing that diabetes can affect vision are other prominent barriers to routine diabetic eye exams [14, 16, 17].

More pervasive barriers to diabetic eye exams also exist, such as fear of negative results, distrust in physicians, forgetfulness, and low self-efficacy [12]. Interventions like educational outreach and reminder messages can help to address some of these barriers to promote diabetic eye exams [18, 19]. We found that the majority of participants in our study were receptive to receiving phone calls or text message reminders to schedule a screening eye appointment. Follow up with mobile communication could be one potential avenue to promote eye exams for ED patients.

Although our study has notable strengths by bringing teleophthalmology for DR screening to the ED setting, our work also has several limitations. The results of our single-center study may not be generalizable to other ED populations. Furthermore, the cohort included in this study may not accurately represent diabetic patients in our ED, as patients were not included if there were factors such as medical instability, active patient care, or inability to sit at the fundus camera due to body habitus or feeling too unwell. Future consideration could be given to comparing the demographic and clinical characteristics of patients eligible for screening with those of patients who are ineligible due to clinical instability or active care. Moreover, experiences from our program could inform the design of future screening interventions. For instance, we found that active clinical care limited patient recruitment from ED examination rooms. As such, future programs may consider recruiting from an earlier stage of the ED encounter, such as from the main ED waiting area before rooming. Additionally, follow-through after screening is critical for an ED-based program to successfully reduce vision loss from DR, and future research would be needed to assess rates of outpatient ophthalmology follow-up. Similarly, future studies should also assess patient satisfaction with an ED-based screening program.

Nonmydriatic fundus photography has its own inherent limitations. Our interpretable image rate of 79%, while comparable to that of other ED-based teleophthalmology programs [20], highlights the challenges of fundus photography in ED settings with uncontrolled lighting conditions and a busy clinical environment. Although commonly used for DR screening, fundus photographs of the macula may miss some cases of DR, such as those with mild peripheral disease. While nonmydriatic, single-field photographs can be taken by nonphysician staff with little training [20], the costs for implementing DR screening in the ED would require institutional investment. Additionally, the store-and-forward approach of acquiring images for remote interpretation by an ophthalmologist limits real-time feedback for patient care or referral. In the future, real-time analysis and artificial intelligence to detect DR from digital photographs may allow for immediate image interpretation, which could risk-stratify urgent referrals for vision-threatening disease [21, 22].

Ultimately, implementation of an ED-based teleophthalmology screening DR program would require buy-in from multiple parties (payors, ED nursing staff, interpreting ophthalmologists), and further research is warranted on its feasability [23]. Our study suggests that teleophthalmology for DR screening in select patients can be a tool to diagnose previously undetected DR among ED patients.

## Conclusions

Fundus photography in the ED identified a high prevalence of previously undiagnosed DR. Over half of participants surveyed reported barriers to routine eye care. Some of these barriers, such as feeling vision is fine or feeling too busy for an eye exam, may be mitigated with reminder messages for routine screening. Future studies are warranted to assess scalability of ED-based screening programs and their follow-through rates.

## Data Availability

The datasets used and/or analyzed during the current study are available from the corresponding author on reasonable request. The authors do not wish to share the dataset in a public repository due to ongoing follow-up analyses.

## Abbreviations

DR: Diabetic retinopathy
ED: Emergency department
HbA1c: Hemoglobin A1c
